# A path analysis study of retention of healthcare professionals in urban India using health information technology

**DOI:** 10.1186/s12960-015-0055-x

**Published:** 2015-07-31

**Authors:** Indrajit Bhattacharya, Anandhi Ramachandran

**Affiliations:** 1 Centre for Health Informatics of National Health Portal, National Institute of Health and Family Welfare, Gangnath Marg, Munirka, New Delhi 110067 India; 2 International Institute of Health Management Research, Plot No. 3, Sector 18A, Dwarka, New Delhi 110075 India

**Keywords:** Healthcare information technology (HIT), Job satisfaction, Intention to stay (ITS), Healthcare professionals, Retention strategy, Path analysis model

## Abstract

**Background:**

Healthcare information technology (HIT) applications are being ubiquitously adopted globally and have been indicated to have effects on certain dimensions of recruitment and retention of healthcare professionals. Retention of healthcare professionals is affected by their job satisfaction (JS), commitment to the organization and intention to stay (ITS) that are interlinked with each other and influenced by many factors related to job, personal, organization, etc. The objectives of the current study were to determine if HIT was one among the factors and, if so, propose a probable retention model that incorporates implementation and use of HIT as a strategy.

**Methods:**

This was a cross-sectional survey study covering 20 hospitals from urban areas of India. The sample (*n* = 586) consisted of doctors, nurses, paramedics and hospital administrators. Data was collected through a structured questionnaire. Factors affecting job satisfaction were determined. Technology acceptance by the healthcare professionals was also determined. Interactions between the factors were predicted using a path analysis model.

**Results:**

The overall satisfaction rate of the respondents was 51 %. Based on factor analysis method, 10 factors were identified for JS and 9 factors for ITS. Availability and use of information technology was one factor that affected JS. The need for implementing technology influenced ITS through work environment and career growth. Also, the study indicated that nearly 70 % of the respondents had awareness of HIT, but only 40 % used them. The importance of providing training for HIT applications was stressed by many respondents.

**Conclusion:**

The results are in agreement with literature studies exploring job satisfaction and retention among healthcare professionals. Our study documented a relatively medium level of job satisfaction among the healthcare professionals in the urban area. Information technology was found to be one among the factors that can plausibly influence their job satisfaction and intention to stay. Based on the results of the study, a retention strategy has been suggested that utilizes implementation of HIT and provision of training to influence the retention of healthcare workers.

## Background

The healthcare sector, especially the hospital industry, is a service-intensive industry that relies heavily on advanced medical technology and availability of trained healthcare professionals for delivering quality healthcare services [1–3]. Among the challenges facing the industry is the high turnover (attrition) of healthcare professionals leading to a global shortage of nearly 7.2 million healthcare workers that is expected to increase to 12.9 million by 2035 [4]. The shortage of skilled workers in hospitals has led to sub-optimal levels of patient care, increased patient mortality, increased medical errors, etc. [5, 6]. Better remuneration, ideal working conditions abroad, high-stress levels, increased workload and reduced job satisfaction (JS) are some of the significant factors identified causing turnover in healthcare professionals [7, 8]. Consequently, this has reportedly led to the exodus of healthcare workers from developing countries to places where their expectations are met. Recruiting and retaining these knowledge professionals is fast becoming a point of concern for all hospitals.

India is one of the fastest growing economies in the world in terms of GDP and is expected to be the third largest economy by 2050 [9]. With the Indian healthcare industry experiencing phenomenal growth, hospitals are moving towards excellence rather than survival and are gearing up to fulfil the gaps in three key areas of importance, that is, people, process and technology. Still, there are many challenges to be overcome, in terms of major out-of-pocket healthcare expenditure, changing lifestyles, varied demographics, infrastructure shortages and widespread inaccessible geographical locations. A major roadblock facing the industry is the shortage of healthcare professionals blown to epidemic proportions, rampant among both public and private sectors. India’s ratio of 0.7 doctors and 1.5 nurses per 1000 people is dramatically lower than the WHO average of 2.5 doctors and 3.0 nurses per 1000 people [10]. Furthermore, there is an acute shortage of paramedical and administrative professionals. The situation is further aggravated by the concentration of medical professionals in urban areas that have only 30 % of the country’s population in comparison to rural areas. There is an additional need of 1.54 million doctors and 2.4 million nurses to match the global average [11]. To deal with these constraints, hospitals have been adopting retention strategies like offering higher pay, job autonomy, higher educational plans, fellowships abroad, work recognition and inclusion of family in social/recreational activities [12–15]. There are no particular strategy/strategies that have been found to be highly successful so far.

In the current global healthcare scenario, information and communication technology (ICT) in the form of healthcare information technology (HIT) applications are perceived as a transformative force and widely adopted for improvement of healthcare service delivery and hospital-wide performance efficiency [16]. The Indian healthcare industry is growing at a tremendous pace due to its strengthening coverage, services and increasing expenditure by public as well as private players. The Indian hospital sector is poised to grow to US$ 280 billion by 2020 [17]. Despite India being an IT-enabled services behemoth, the use of HIT is very restricted in the country. Huge private sector investments in HIT are on the increase [18, 19] which includes big pharmaceutical companies, corporate hospitals and other private health sector institutions, while the public healthcare sector is lagging way behind in IT utilization. To bring a paradigm shift in the healthcare dynamics in the country, the public healthcare sector is zealously pursuing implementation of information technology tools in the country. The Indian government has initiated a dreams project, namely the “National Optic Fibre Network (NOFN)” – enabling broadband connectivity to 250 000 panchayats; “Digital India” – envisaging transformation of India into a digitally empowered society and knowledge economy by adopting technology for poverty reduction, curtailing healthcare cost burden and provision of quality healthcare services; and “building Smart cites” – enabling the use of digital technologies or ICTs to enhance quality and performance of urban services, including e-health, to reduce costs and resource consumption and to engage more effectively and actively with its citizens [20–24]. As a part of the project, it has rolled out its strategy in enabling widespread implementation and adoption of HIT through electronic medical records (EMRs), telemedicine, online medical consultation and medicine supply and m-health initiatives [23, 24].

Adoption of technology is known not only to reduce medical errors, decrease work load and increase patient care and safety [25, 26] besides increasing complexity and initial work load at the same time [27]. It has been demonstrated in several instances that it may have a positive influence on the recruitment and retention of healthcare professionals in rural and remote regions [28–31] where technologies like tele-health and videoconferencing are in practice. There are also reports indicating that ICT has no effects on retention [32, 33]. Retention studies related to ICTs in urban areas are few. Gagnon et al [34] in a systematic review article have explored these aspects and have highlighted the necessity of further studies exploring the dynamics between retention and various personal, professional, educational and organizational factors. Many HIT implementations have failed as they majorly focus on solving clinical problems and simplifying the administrative processes instead of echoing the needs of the end users of the applications [35, 36]. The perceptions of benefits and adverse effects of adopting technology in healthcare are linked to the acceptance of the technology by healthcare professionals, their intention and eagerness to adopt them in their work culture. Hence, the factors that can affect the acceptance of technology are required to be explored initially, before developing a plausible retention strategy.

This manuscript is based on the study carried out in the urban areas of northern and central parts of India, to explore the possibility of using HIT implementation as one of the probable strategies to reduce retention. The approach adopted is that turnover (attrition) among healthcare professionals is influenced by two dominant variables, the JS felt by the healthcare professionals and their intention to stay (ITS) in the job. JS is the degree to which individuals like (satisfaction) or dislike (dissatisfaction) their job [37, 38]. It indicates the attitudinal component of the attrition. “Intention to stay” is indicative of the decisional aspect and reflects the keenness or interest of the employees to continue in the organization. It is assumed that attitude influences the decision taken by the professionals which results in turnover. The resulting turnover is the actual behavioural process. These components can be best understood by focusing on the interrelated facets or dimensions that in turn influence them.

In a recent unpublished study exploring the HIT landscape among hospitals in the capital of Delhi, it was identified that though nearly 80 % of corporate hospitals and public hospitals have adopted use of technology as part of daily work practices [39], it has been adopted to a minimal level to none among small (less than 100 beds) and medium hospitals (less than 300 beds). Due to migration of the rural population to urban and semi-urban areas in search of livelihood, lack of basic facilities, infrastructure and trained professionals in the rural areas, there is an increased footfall of patients in urban areas. There are reports from urban hospitals indicating high attrition among their physicians and nurses and various strategies being adopted for their retention by the management [40, 41]. In line with this, the present study explored whether the need of HIT application and its availability were indicated as one of the factors of JS and ITS, respectively. Further, it was probed whether the lack of availability of HIT applications was indicated by the professionals to be one of the reasons for shifting jobs. The research questions asked in the study were, “What are the major factors affecting JS and ITS among healthcare professionals in urban settings?”, “Does availability of HIT applications affect JS and ITS?”, “Can implementation of HIT be suggested to be a adopted as a probable retention strategy?” and “What is the technology acceptance among healthcare professionals?” The main focus was to identify the factors affecting job satisfaction and intention to stay and explore whether HIT can be suggested as one among those factors affecting JS and ITS. Hence, the current study targeted small-to-medium hospitals to study the influence of HIT implementation for retention.

## Methods

### Study design and settings

The study design adopted was descriptive using cross-sectional data collected from public and private hospitals in the urban locations of northern and central parts of India. All the hospitals, multi-speciality nursing homes in and around major cities with a minimum 50 beds and providing tertiary services, were invited to participate in the survey. It was presumed that huge patient inflow would necessitate employing a large number of healthcare professionals, thereby being an ideal platform for studying factors affecting attrition among healthcare professionals. Since the objective of the study was to check if availability and implementation of HIT could be used as one of the strategies for reducing attrition among healthcare professionals, implementation of health information systems (HISs), EMR, etc. was not considered as mandatory criteria for participation of hospitals in the survey. From the list of 36 hospitals who agreed to participate in the study, 20 hospitals were randomly selected. Each hospital was provided a unique ID which was fed into a computer-generated algorithm for the final selection of 10 private and 10 public hospitals. HIS and EMR were available in 13 of the selected hospitals.

### Study participants

The respondents selected were doctors, nurses and administrators, and their selection criteria were based on their Indian resident status, valid registration licence to practise, minimum 1 year of working in the current organization and work experience of minimum 3 years in the industry. This target group was proposed after discussions with experts in the field and also based on a quick scan of the hospitals to identify the frequent and large-scale users of HIT. Pharmacists, lab technicians and paramedics are not included in the current paper.

All the doctors, nurses and administrators who satisfied the selection criteria mentioned earlier were requested to participate in the survey after obtaining approval from the concerned authorities. Finally, 982 respondents were found eligible for the survey.

### Survey instrument

The structured questionnaire was administered to the respondents through direct interaction and through email, assuring confidentiality and anonymity. Filled questionnaires with missing, ambiguous or incomplete responses were not considered for analysis. The entire questionnaire (semi-structured) was designed based on the tools already available for determining job satisfaction, intention to stay [42–45] and HIT technology acceptance [46, 47] and entailed close-ended questions. It was divided into six sections: demographic information, assessment of JS, assessment of ITS in the organization, reasons for leaving earlier job, future plan related to their current job and assessment of technology acceptance in work place. Pre-testing was done with an aim to check the format, language, sequence and comprehension of the questions. It was carried out in two hospitals (one public and one private) within the National Capital Region (NCR) region of Delhi. From both the organizations, respondents were selected with an equal mix of experience, gender, seniority and target group to validate the instrument under the discretion of hospital authority. Incorporating their feedback, the survey instrument was finally modified to have the following number of questions: 7 questions covering demographic and categorical variables (gender, education, marital status, total years of experience, duration of stay in the current organization, type of the organization (public and private), income), 13 questions covering dimensions of JS, 11 questions covering dimensions of ITS and 5 questions covering attributes related to the healthcare professional’s acceptance of technology (knowledge and awareness, existing practice, intent to use, perceived benefits and attitude towards use of HIT in work). Respondents who had changed jobs within the past 1 year of the survey were provided with a list of reasons that generally instigates a healthcare professional to shift the job and were asked to choose the most probable reasons according to their perspective. Similarly, all respondents were questioned whether they were planning to shift jobs within 12 months, and if so, they were asked to list the reasons that are instigating them to do so.

The tool for JS and ITS used was a five-point Likert scale for rating, and the reliability scale of these parts of the tool were 0.84 (Cronbach’s alpha). A seven-point Likert scale was used to measure the individual items contributing to HIT attributes, and the reliability scale of this part of the tool exceeded 0.78.

Some of the study questions were related to personal details of the respondents related to their attitude, behaviour, self-motivation and belief, and hence, it can be expected that the individual responses may depend upon their self-interests. As a case study, secondary data from exit interviews of healthcare professionals (sample size = 45) from one of the hospitals was reviewed. This helped the researchers to verify whether there were other reasons for shifting jobs than those listed in the survey questionnaire.

### Data analysis

Data analysis was conducted using the Statistical Package for the Social Sciences (SPSS Inc., version 18.0, Chicago, IL, USA). The primary unit of analysis in this study was the type of healthcare employee (doctors, nurses and administrators). Bivariate analysis was used to compare responses from participants in each of the employer types, with both descriptive and inferential statistical methods. Chi-square and *t*-tests were conducted to detect levels of significance. The exploratory factor analysis (EFA) on rotated factor matrix using principal analysis factoring (PAF) with varimax rotation was performed to determine the factors independently influencing job satisfaction and intent to stay. The Kaiser-Meyer-Olkin (KMO) measure of sampling adequacy and Bartlett’s test of sphericity were used to assess the suitability of the respondent data for factor analysis [48]. Both scree test and Kaiser’s rule (eigenvalues > 1) were used to extract the number of factors. Individual items (questions) contributing to a particular factor were grouped based on a factor loading of 0.5 or greater. No single item contributed to more than one factor and each factor had minimum three items contributing to it. Once the factors were identified, the relationship between these factors and personal/demographic characteristics were probed via univariate (ANOVA) parametric tests. Where significant interaction effects were observed, the locus of these was determined via chi-square. A correlation coefficient (*r*) analysis was conducted to examine if the variables correlated with each other. Level of significance was set at a *P* value of ≤0.05. Path analysis was carried out using the open-source tool OpenStat V [49]. For determining the path model, the factors identified through EFA and the demographic variables were considered as exogenous variables similar to independent variables, and JS and ITS were considered as endogenous, similar to dependent or outcome variables. Exogenous variables represent those constructs that exert an influence on other constructs under study and are not influenced by other factors in the quantitative model. Those constructs identified as endogenous are affected by exogenous and other endogenous variables in the model [50].

## Results

All the 982 participants who were found eligible as per the selection criteria to participate in the survey were provided the questionnaire to be completed. From the responses received, only 586 responses were found to be eligible for analyses. Table 1 highlights the demographics of the respondents. Men represented 57 % of the respondents. The age group most represented in the survey was 26 to 35 years of age. The maximum age of the respondents observed were 55 years of age. One third of the healthcare professionals had 5–10 years of working experience. The distribution of the respondents in respect to the nature of work was 45 % doctors, 35 % nurses and paramedics and remaining 20 % administrators. Most of the respondents were graduates (54.3 %). Nearly two thirds of the respondents were married and living as couple. Healthcare professionals with income in the range of 20K to 30K participated at a higher proportion. The proportion of respondents who participated from the private hospitals was greater (61 %) than public hospitals.

**Table 1:** Demographic detail of the respondents

Details		*N* = 586	%
Gender	:Male	334	57
	:Female	252	43
Marital status	:Married	363	62
	:Unmarried	223	38
Age	:17–25 years	108	18.5
	:25–35 years	303	51.7
	:36+ years	175	29.8
Education	:Undergraduate	67	11.5
	:Graduate	318	54.3
	:Postgraduate	200	34.2
Nature of work	:Doctors	264	45
	:Nurses, paramedics	205	35
	:Administrators	117	20
Income (Rs)	:Up to 10K	123	21
	:10K–20K	117	20
	:20K–30K	152	26
	:30K–40K	94	16
	>40K	100	17
Type of hospital	:Public	217	37
	:Private	363	62
Work experience	:<5 years	135	23
	:5–10 years	211	36
	:10–15 years	117	20
	:15–20 years	70	12
	:>20 years	53	9

The respondents were asked to rate themselves on the level of satisfaction they felt towards their job. Of the respondents, 76 % rated themselves to be overall satisfied with their job. Among the respondents, 18.7 % were planning to shift anytime within a year if opportunity arose and another 14.9 % within next 2 years. Nearly two thirds were doctors and the rest were nurses and administrators. Based on the self-rated satisfaction level and plan to shift jobs, the proportion of respondents actually dissatisfied with their job was recalculated to 49 %. The doctors were more dissatisfied than the nurses and administrators. The doctors working in private hospitals (37 %) were more dissatisfied than those working in public hospitals (nearly 20 %).

Based on factor analysis, 10 factors were identified as main contributors of JS out of the 13 dimensions surveyed (Table 2). They were compensation and perks, work–life balance, sense of accomplishment, imbalance of workload, need for automation technology, substandard nature of work, autonomy and security, relationship with peers, work satisfaction and organization influence. The respondents were much less satisfied with their salaries (50 %), work–life balance (38 %) and sense of accomplishment (26 %).

**Table 2:** Factor analysis values for job satisfaction

Job satisfaction			
Factor	Item(s) considered	Factor loading	Factor name
1	• Salary and financial benefits	• 0.6814	Compensation and perks
	• Non-financial incentives	• 0.6482	
2	• Policies related to employees	• 0.6699	Work–life balance
	• Facilities for employee comfort	• 0.6796	
	• Work facilitation	• 0.6586	
3	• Self-achievement and satisfaction	• 0.7341	Sense of accomplishment
	• Self-esteem	• 0.7186	
	• Freedom in job	• 0.6187	
4	• Work overload	• 0.8540	Imbalanced work load
	• Exhaustion from work	• 0.6086	
	• Work stress	• 0.6145	
5	• Innovation through automation	• 0.8383	Need for technology implementation
	• Information technology requirement	• 0.6815	
	• Technical support of IT	• 0.7904	
6	• Interesting and motivating	• 0.9062	Substandard nature of work
	• Challenge	• 0.6788	
	• Skill variety	• 0.7142	
7	• Job security	• 0.6771	Autonomy and security
	• Responsibilities	• 0.6989	
	• Decision making	• 0.7704	
8	• Cooperation	• 0.6980	Relationship with peers
	• Trust and support	• 0.6218	
	• Amicable behaviour	• 0.7002	
	• Conflict	• 0.6689	
9	• Justice	• 0.6072	Work satisfaction
	• Recognition	• 0.6220	
	• Role clarity	• 0.6139	
	• Career growth	• 0.6058	
10	• Pride	• 0.6551	Organization influence
	• Organization experience	• 0.6427	
	• Organization identity	• 0.6405	
	• Emphasis on quality	• 0.6315	

**Fig. 1:**
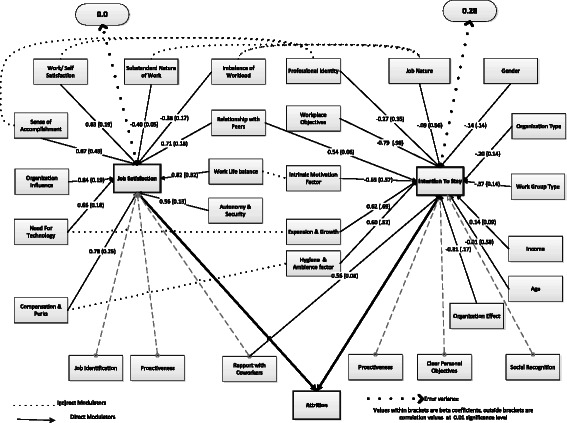
Path model depicting the various modulators affecting both the outcome variables

A multi-group path analysis was constructed to generate a model for overall JS (Fig. 1). Every path identified in this path analysis was statistically significant (*P* < 0.05). Based on the adjusted goodness of fitness index (AGFI = 0.905), comparative fit index (CFI = 0.910) and root mean square error of approximation (RMSEA = 0.048), a best fit model was selected. In this model, the identified factors that contributed to JS were considered as direct modulators. Based on the path coefficients (*β*), “sense of accomplishment” (*β* = 0.49) and “work–life balance” (*β* = 0.32) were the maximum contributors followed by “compensation and perks” (*β* = 0.25) and “need for technology” (*β* = 0.21). All the factors correlated well with JS and few among themselves. “Sense of accomplishment” had greater correlation with “work–life balance” (*r* = 0.66), with “work satisfaction” (*r* = 0.75), with “organization influence” (*r* = 0.78) and with “need for information technology” (*r* = 0.63). The three dimensions that did not contribute to factors of JS, that is, “pro-activeness”, “rapport with co-workers” and “job identification” correlated significantly with each other and with some of the direct modulators. They were the indirect modulators of JS. “Rapport with co-workers” correlated with direct modulators “relationship with peers” and “imbalanced work load” (*r* > 0.5 in both cases). “Job identification” correlated well with three direct modulators, “work satisfaction” (*r* = 0.41), “sense of accomplishment” (*r* = 0.34) and “security and autonomy” (*r* = 0.31). “Pro-activeness” was a single item, and its correlation to sense of accomplishment was *r* = 0.38.

Nine factors were extracted for ITS (factor loadings > 0.5) based on EFA (Table 3), which is indicative of the respondents’ motivation and commitment to the job. They were financial and non-financial rewards (grouped as hygiene and ambience factors), intrinsic motivation factors, professional identity (self-esteem, accomplishment), nature of job, organization effect, expansion and growth, rapport with co-workers, workplace objectives and relationship with peers. Among these, five factors, namely financial and non-financial rewards, professional identity nature of job, relationship with peers and co-workers, were also factors contributing to JS (Table 2).

**Table 3:** Factor analysis values for intention to stay

Intention to stay			
Factor	Item(s) considered	Factor loading	Factor name
1	• Provision of amenities like housing, conveyance, medical benefits.	• 0.6081	• Hygiene and ambience factor
	• Facilities provided for effective working	• 0.6727	
	• Security	• 0.6373	
2	• Acceptable working schedule	• 0.6656	• Intrinsic motivation factor
	• Opportunity for personal growth	• 0.5578	
	• Authority	• 0.5440	
	• Opportunity for career advancement	• 0.6245	
3	• Recognition	• 0.6355	• Professional identity
	• Opportunity to become well-known and well-off.	• 0.6368	
	• Self-esteem and feeling satisfied	• 0.5916	
	• Prestige of the job outside in industry	• 0.5716	
	• Job identification to professional goals	• 0.5086	
4	• Interesting and motivating	• 0.6001	• Job nature
	• Learning environment	• 0.6168	
	• Less of stress	• 0.7716	
5	• Trust and support	• 0.5260	• Organization effect
	• Friendly environment	• 0.5624	
	• Effective leadership	• 0.5988	
6	• Opportunity/promotion	• 0.6100	• Expansion and growth
	• Technology for skill development	• 0.5987	
	• Innovation	• 0.5880	
	• Training environment	• 0.5011	
7	• Trustworthy co-workers	• 0.6231	• Rapport with co-workers
	• Colleague support	• 0.7054	
	• Relationship with co-workers	• 0.7883	
8	• Objectives of workplace	• 0.5012	• Workplace objectives
	• Purpose of nature of job	• 0.6500	
9	• Cooperation	• 0.6790	• Relationship with peers
	• Trust and support	• 0.6120	
	• Amicable behaviour	• 0.6656	
	• Knowledge sharing	• 0.6345	

Path analysis was also performed to determine an individual model for ITS. Three items that did not contribute to factor analysis: “pro-activeness”, “clear personal objectives” and “social recognition”, were considered as indirect modulators. The rest were considered as direct modulators. Based on the initial model, AGFI = 0.78 with error variance 0.53 was obtained which indicated that there are still factors contributing to ITS other than those accounted. Hence, the model was recalculated by including both JS and demographic variables as direct modulators. This produced a path model with AGFI = 0.92, CFI = 0.90 and RMSEA = 0.45. The error variance was reduced to 0.28. While few variables like gender, age, workgroup type and organization type had negative correlation with ITS, income had a small positive correlation. Marital status and education did not contribute to path models. Among the direct modulators of ITS, “work place objectives” (*β* = 0.98) and “expansion and growth” (*β* = 0.69) were significant, followed by “hygiene and ambience factors” (*β* = 0.52), “nature of job” (*β* = 0.56) and “intrinsic motivation” (*β* = 0.57). Among the demographic variables, age seemed to be the significant contributor (*β* = 0.58). Among the direct modulators, the co-relation was higher (*r* > 0.35). Since the ITS path model contained factors that influenced JS, the final path model depicts an interlinked path model for both the outcome variables JS and ITS (Fig. 1).

**Fig. 2:**
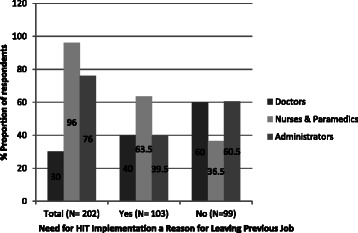
Distribution of respondents who shifted within past 1 year

**Fig. 3:**
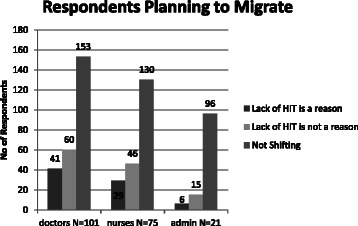
Distribution of respondents who are planning to shift within 2 years

The reasons perceived by the healthcare professionals for leaving their previous organization were sought on the basis that it would throw light on the factors that affect the JS and also could aid in developing retention strategy for the professionals. Nine reasons were opted by the respondents; they are heavy workload, non-cooperative boss, lack of incentives, absence of social security benefits, low-pay structure/compensation, lack of on-the-job training, frequent transfers, lack of new skill development and absence of technology in their work. Except frequent transfers and absence of social security, all other reasons were rated highly by the respondents. Similarly, respondents who were planning to leave the current organization were asked to provide the factors or reasons influencing their decision to migrate. They were as follows: opportunity to upgrade qualifications, skills and use of newer technologies, absence of amicable working environment, better remuneration, possibility of career advancement and social and professional recognition. Among the respondents who had shifted from their previous job within a year, nearly 51 % of the respondents indicated that the lack of HIT application was one of the reasons for shifting job. Nurses (63.5 %) provided this as a major reason compared to other respondents in greater proportion (Fig. 2). Nearly 33.6 % of healthcare professionals were planning to shift within next 2 years if there was an opportunity. One of the reasons provided by them was the lack of technology in their work environment to reduce their workload (Fig. 3).

**Fig. 4:**
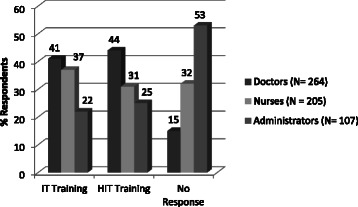
Distribution of respondents against need for IT and HIT training

Among the respondents, 81.7 % doctors, 76.8 % nurses and 71.2 % administrative staff had basic awareness of computers and their usage. From the survey, it could be identified that 79.8 % administrative staff, 33.3 % nurses and 37.2 % doctors utilized information technology for work via email, HIS, video conferencing, etc. Percentage of IT usage was higher among the age bracket of 26–35 years than other age groups. Doctors (41 %) felt the benefits of using technology more than nurses (38 %) and administrators (22 %). The mandatory need for HIT implementation for improving quality of services was reported by 45 % doctors, 31 % nurses and 25 % administrators. The existing practice of using HIT in the form of EMR, computerized physician order entry (CPOE), etc. were analysed only among the respondents (*N* = 371) in those hospitals where HIS and EMR have been implemented. Among them, the keenness (intent) to use technology and perceived realization of the benefits of using technology was less among doctors (39 %) when compared to nurses (46 %). Among the respondents from those hospitals where HIT was not yet implemented (*N* = 216), nearly 75 % of the administrators showed keen interest in using HIT for generating resource utilization reports, getting patient-related information, appointment and scheduling, asset management, etc. if made available. Correlation analysis among the factors affecting technology acceptance (Table 4) resulted in greater correlation between attitude and technology acceptance, ease of use and technology acceptance. Correlation analysis resulted in perceived benefits (*r* = 0.720) and attitude (*r* = 0.718) having higher correlation to acceptance of technology (Table 4). Similarly, perceived benefits were highly correlated with attitude (*r* = 0.667) and basic awareness of technology (*r* = 0.624). Basic awareness also influenced attitude (*r* = 0.512). All the respondents were queried on the necessity of providing training in basic technology and HIT applications (Fig. 4).

**Table 4:** Correlation values between various HIT attributes

Hospitals where HIT is implemented (r values)					
Factors affecting Technology Acceptance	Perceived benefits	Existing practices	Ease of use	Attitude	Acceptance of technology
Perceived benefits	1				
Existing practices	0.6140	1			
Ease of use	0.4311	0.4681	1		
Attitude	0.5671	0.4123	0.5910	1	
Acceptance of technology	0.5200	0.4645	0.6216	0.7181	1
Hospitals where HIT is not implemented (r values)					
Factors affecting Technology Acceptance	Perceived benefits	Basic awareness	Openness for HIS	Attitude	Acceptance of technology
Perceived benefits	1				
Basic awareness	0.6239	1			
Openness	0.3271	0.2681	1		
Attitude	0.5769	0.5123	0.4910	1	
Acceptance of technology	0.5500	0.4645	0.4181	0.6945	1

The review of the exit interviews (secondary data) identified that the overall causes for leaving the job as cited by the past employees in the forms were inadequate remuneration, increased workload due to understaffed employees and lack of professional advancement. Skewed ratings were observed for interpersonal relationships between superiors, peers and subordinates among doctors, nurses and administration. From the data, it could be observed that the reasons indicated for leaving jobs were higher education and specialization by graduate doctors and attractive salary packages and chance for super-specialization by postgraduate doctors. Although the implementation of HIS in the hospitals was lauded, it was not felt effective enough for their professional growth. Most of the nurses had mentioned in their exit interviews that they had work overload due to maintenance of many registers. Most of the doctors had stated in the interview that they were open to come back for serving in the hospital. The nurses and administration staff were less willing to come back even on a higher pay package.

## Discussion

The Indian healthcare system is a mixture of both public and private hospitals with heavy out-of-pocket expenditure being more than 70 % [51]. The organization or working environment can have a profound effect on the quality and cost of the services provided [52]. So, it is imperative for organizations to understand the factors that enable their employees to achieve the highest level of JS. Though the majority of the participants had rated themselves as satisfied with their job (highly and moderately satisfied), many had indicated their plan to shift jobs within a year if opportunity arises. This indicates a deep-down dissatisfaction in the job and with their organization. Such dissatisfaction among health professionals is a cause for concern since JS is known to affect the overall efficiency, performance, absenteeism and turnover [53, 54].

In the current study, the doctors working in private hospitals were more dissatisfied than those working in public hospitals in spite of the higher salary packages and benefits. This might be due to the heavy work-load-related pressures. Overall, the results showed marked variation in levels of satisfaction from one dimension to another. Compensation and perks refers to the availability of incentives and extra financial benefits to the healthcare service workforce. There are reports that provide such examples where the use of provider incentives and enablers has known to increase the performance under certain conditions [55, 56]. Paying incentives to reduce attrition involves crafting proper incentives, developing indicators of activity to be measured, implementing them and monitoring the performances on a timely basis by the organization that may cause operational difficulties. While developing incentives, non-financial fringe benefits should also be considered [15].

Work–life balance was one among the factors affecting JS in the current study. There are earlier literature reports stating that low satisfaction of the employees with salaries, non-participation in decision making, routine mundane tasks and insufficient work–life balance can cause a decrease in JS [57–59]. Work–life balance is about helping employees better manage their work and personal (non-work) time. This refers to family-friendly work arrangements and alternative work arrangements. It depends on the nature of the work and workplace and issues and policies prevailing in the workplace. Introducing strategies like flexible work options, specialized leave policies, paid maternal leave, paternal leave and home telecommuting can increase the satisfaction level of the healthcare professionals.

Hospitals in the urban areas cater to the nearby rural area too where there is a dearth of specialized services. Moreover, with public and private insurance agencies increasing their spectrum of services to cover many chronic diseases, there is a growing inflow of patients to both public and private hospitals in urban areas. All these have caused an increased work load to the healthcare professionals. Peer relationship was identified as another significant factor to JS. Nurse–physician relationships, disruptive physician behaviour and institutional responses to such behaviour, all have impact on nurses’ job satisfaction, morale and retention [60]. Although only a small percentage of healthcare professionals reported occurrence to exhibit disruptive behaviour, both physicians and nurses agreed that it influences nurses’ as well as other staff members’ attitudes towards patient care and inhibits teamwork affecting the efficiency, accuracy, safety and outcomes of care.

Sense of achievement and fulfilment was indicated as one of the factors contributing to JS in this study. This depends on the oneness felt by the employees with the organization and is influenced by their compatibility towards the organization’s mission, vision, goals and strategies. The work satisfaction felt by the health professional relates to the sense of role clarity and recognition given to the work. Morrison et al. [61] outlined several ways in which lack of engagement and high-turnover rates impact healthcare organizations. Some of these factors include turnover costs, which according to Waldman and Kelly [62] range between 3.4 % and 5.8 % of their operating budget. When employees feel dissatisfied and not recognized, they leave the organization and this puts higher workloads and stress levels on those who remain and ultimately is further driving down satisfaction for both employees and patients [63]. Employees’ needs and motivators vary, so it is important to understand what motivates them to perform [64, 65]. The conditions under which healthcare professionals work can have as much impact on their effectiveness, comfort and safety as the intrinsic details of the task itself.

Professional autonomy motivates employees to show commitment to their organization, enhancing work conditions to support the organization’s mission and thus impacting on JS [66]. Similarly, organizational support improves work satisfaction and diminishes the turnover rate among physicians [67]. Numerous studies conducted among healthcare professionals point to the importance of the interpersonal relationships in JS [68] and that it is likely to increase the client safety, improved quality of care and greater client satisfaction. But in the current study, the contribution of interpersonal relationships does not contribute as a direct modulator of JS. We believe it must be due to the fact that given the work load and busy nature of the professionals due importance has not been given to this question by the respondents.

A healthcare professional’s ITS is affected by the interplay of complex factors at the organizational, managerial and personnel level. In a number of earlier research findings, most of the variance of ITS was consistently explained by JS [69, 70]. The path model identified that the JS dimensions, financial and non-financial rewards, professional identity, nature of job and relationship with peers and co-workers, affect ITS also. At the personal level, remuneration is a critical factor of motivation and retention [71]. A high proportion of healthcare professionals particularly from public hospitals feel that their remuneration is not fair. Opportunities for promotion or career growth, self-esteem and accomplishment are other key dimensions of ITS [72]. Socio-demographic factors like gender, age, education, organization type and experience have been known to have influence on ITS [73]. In the current study, gender, education and organization type affected ITS. Females were likely to pursue for longer duration than males. The graduates were keen on continuing compared to postgraduate professionals. This may be due to the fact that the latter were more oriented towards better remuneration, professional identity and expansion and career growth. Expansion and career growth indicated learning newer skills like use of HIT, promotion and shifting to newer domain areas. Such influences have been reported earlier in studies related to nurses [71, 74].

We also found that healthcare professionals’ assessment of the adequacy of their support from their organization influenced their intention to remain employed [75]. Organization support, its culture, values and beliefs, affects a healthcare professional’s oneness with the organization. This depends upon the extent to which the organization is able to convey to the healthcare professional a sense that he/she is a valued employee through its words and actions. Working professionals remain dissatisfied with their organization if they feel they are not getting adequate support from the management [76].

Need for HIT implementation was indicated as one of the factors affecting JS. Similarly, the availability of HIT was highlighted as the factor affecting their ITS. Most of the respondents irrespective of gender, age and education felt that implementation of health information technology in day-to-day work life was necessary. Many respondents who were surveyed felt that use of technology will provide skill enhancement and keep them abreast with the newer developments in the industry. It is believed that HIT applications like HIS and EMR help to improve efficiencies and bring in innovations in work, reduce workload and can increase their quality performance. In concordance with this opinion, it could be seen that one of the reasons provided by the professionals planning to leave the organization was the lack of technology in their work. The healthcare industry is increasingly influenced by information technology, be it use of sophisticated medical devices, communication devices (mobiles, smart phones), telemedicine or longitudinal patient record maintenance through EMRs and clinical decision systems [77–81]. It is known to help professionals to confidently and securely access, interpret, apply organizational knowledge, patient care procedures, best practices and other skills in a manner that improves patient satisfaction, achieves positive clinical outcomes and maximizes the cost savings for the organization [25, 26].

The nature of work done by respondents (doctors, nurses and administrators) seems to play a significant role in expressing the lack of automation and technology as one of the major factors of attrition. Among the healthcare professionals surveyed, doctors though aware of the benefits of HIT, particularly to patient safety and performance improvement, surprisingly preferred using HIT personally to a lesser extent in their work. On further probing, it was brought to light that it was difficult for them to work with the complex HIT applications especially when they have a busy schedule. But, they felt that technology was much needed in their work and were open to use the help of their juniors or assistants for handling technology. The nurses and administrators were more open to use the technology. But many nurses indicated that dual work of maintaining records both through HIT application and paper increases their workload and hence the slight opposition to large-scale adoption of technology.

Basic awareness and knowledge about HIT applications influenced the benefits perceived by the professionals on using the applications and also their attitude towards acceptance of technology. Once HIT was implemented and the professionals gained an awareness regarding the HIT applications and their benefits, it was their attitude towards technology and the ease of use of the applications that seem to affect the overall technology acceptance. It is well-known that education and training opportunities have strong motivating effects [82, 83]. Training enables workers to take on more demanding duties and to achieve personal goals of professional advancement [84] as well as allow them to cope better with the requirements of their job and are especially important for young health professionals [85]. The need for upgrading skills and use of technologies as a path to career advancement was highlighted to be significant by the respondents. The doctors and nurses were inclined to undergo training if provided compared to the administrators. This might be due to the fact that the former use HIS and EMR for updating the patient data. These applications are complex, and the users need to be trained to use them effectively. Administrators are mostly involved in report generation to a greater extent. These are already provided as readymade templates and are much easier to be worked upon.

## Limitations

Many of the potential participants (nearly 15 %) did not complete the survey due to sudden shifting of jobs or long leaves of absence from work. Some of the senior physicians were not eager to share their personal details like income and their intent to use HIT and gave reasons of busy schedule for not completing the survey. There were ambiguous filling of questionnaire, that is, more than one option was selected for a given item by few respondents (nearly 5 %). These constraints could be seen in the survey conducted mostly through email and to a lesser amount in direct interview. All these resulted in only 60 % of responses being finally found eligible for data analysis.

Given the sampling procedure adopted, as well as the final small-sample size, the results obtained are not claimed to be representative for the entire health workforce of a country. Also, not all hospitals where the survey was conducted had HIT implementation. Hence, it was difficult to explore the linkage between implementation of HIT and job satisfaction in all hospitals.

Despite these limitations and considering the fact that there is a paucity of published evidence relating HIT and retention in India, this study can be used to explore if HIT implementation can be used as a strategy for retention. It would provide some baseline data for future comparison and may trigger an initiative of looking into use of information technology as a possible factor for job satisfaction and retention of healthcare providers.

## Conclusions

**Fig. 5:**
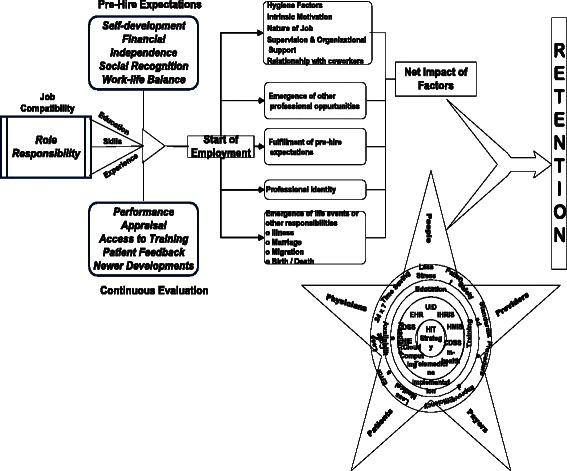
Strategy using HIT for increased retention

The healthcare sector is witnessing the highest attrition rates making retention of critical manpower resources a key challenge. Any effort to strengthen healthcare service quality must concentrate on building and promoting the professional culture of healthcare professionals and should create a conducive environment for working that meets both professional and organization goals. This will increase the job satisfaction, commitment of the professionals towards their organization and promote their intention to stay. The healthcare industry is increasingly influenced by the use of information technology in various forms from record maintenance to patient monitoring, communication and treatments. They can enable healthcare professionals to confidently access, interpret and apply organizational knowledge, patient care procedures, best practices and other skills in a manner that improves patient satisfaction, achieves positive clinical outcomes and maximizes cost savings for the organization. HIT is viewed by healthcare professionals to promote professional ethos and commitment and to strengthen their perception of self-efficacy. But, they cannot compensate for many other factors prevalent at both the macro and micro level, which seriously impinge staff retention, such as staff and supply shortages, difficult working conditions and migration pull factors originating from developed countries. We suggest the following can be utilized as potential strategies that utilize HIT as a component to increase retention among healthcare professionals as illustrated in Fig. 5.

- Healthcare professionals based upon the education, experience and skill set should be assigned a role and allocated individual responsibilities that satisfy their pre-hire expectations in accordance with the industry standards and organizational policies.
- After hiring, a part of orientation training related to the use of HIT applications needs to be provided at levels appropriate to the work requirement.
- This should be backed with a periodic appraisal of the work, improvement in salaries and benefits and upgradation of knowledge and skills.
- When HIT is being adopted for the first time in the organization or changes brought to the existing technology, healthcare professionals should be made aware of management’s objectives for adopting the technology, its benefits to the professional’s work life, patient care and expectations of the management from the staff in using the technology.
- Implementation of HIT applications and using it as a part of the retention strategy will be successful only if the healthcare professionals are open and ready to accept technology in their work environment. Involving the healthcare professionals like doctors or nurses during the designing and implementation will boost the morale of the professionals and give an impetus to adopt easily. A champion among the healthcare professionals who will act as an evangelist among the peers and colleagues will help in bringing about the transformation smoothly.
- HIT implementation should also be used for providing a continuous teaching and learning environment to upgrade knowledge, for discussion with peers and colleagues and as a media for maintaining the communication with patients other than for administrative and performance goals.

Using HIT in a fashion to produce a relatively mild change in the workflow that results in improvement in the service experienced by the patients would hardly be constituted as a disruptive threat to the existing organizational culture by the healthcare professionals. The changes brought about should be a precursor for continual evolution of improvement. This can happen only if implementation of HIT is brought about as a collaborative process and not driven by one cohort or group. In conclusion, we suggest that HIT can be integrated with other retention strategies adopted by the organization to reduce turnover and increase retention.
